# Metabolites of the Polycyclic Aromatic Hydrocarbon Phenanthrene in the Urine of Cigarette Smokers from Five Ethnic Groups with Differing Risks for Lung Cancer

**DOI:** 10.1371/journal.pone.0156203

**Published:** 2016-06-08

**Authors:** Yesha M. Patel, Sungshim L. Park, Steven G. Carmella, Viviana Paiano, Natalie Olvera, Daniel O. Stram, Christopher A. Haiman, Loic Le Marchand, Stephen S. Hecht

**Affiliations:** 1 Department of Preventive Medicine, Norris Comprehensive Cancer Center, Keck School of Medicine, University of Southern California, Los Angeles, CA, 90032, United States of America; 2 Masonic Cancer Center, University of Minnesota, Minneapolis, MN, 55455, United States of America; 3 Epidemiology Program, University of Hawaii Cancer Center, Honolulu, HI, 96813, United States of America; New York University School of Medicine, UNITED STATES

## Abstract

Results from the Multiethnic Cohort Study demonstrated significant differences in lung cancer risk among cigarette smokers from five different ethnic/racial groups. For the same number of cigarettes smoked, and particularly among light smokers, African Americans and Native Hawaiians had the highest risk for lung cancer, Whites had intermediate risk, while Latinos and Japanese Americans had the lowest risk. We analyzed urine samples from 331–709 participants from each ethnic group in this study for metabolites of phenanthrene, a surrogate for carcinogenic polycyclic aromatic hydrocarbon exposure. Consistent with their lung cancer risk and our previous studies of several other carcinogens and toxicants of cigarette smoke, African Americans had significantly (p<0.0001) higher median levels of the two phenanthrene metabolites 3-hydroxyphenanthrene (3-PheOH, 0.931 pmol/ml) and phenanthrene tetraol (PheT, 1.13 pmol/ml) than Whites (3-PheOH, 0.697 pmol/ml; PheT, 0.853 pmol/ml) while Japanese-Americans had significantly (p = 0.002) lower levels of 3-PheOH (0.621 pmol/ml) than Whites. PheT levels (0.838 pmol/ml) in Japanese-Americans were not different from those of Whites. These results are mainly consistent with the lung cancer risk of these three groups, but the results for Native Hawaiians and Latinos were more complex. We also carried out a genome wide association study in search of factors that could influence PheT and 3-PheOH levels. Deletion of *GSTT1* explained 2.2% of the variability in PheT, while the strongest association, rs5751777 (p = 1.8x10^-62^) in the *GSTT2* gene, explained 7.7% of the variability in PheT. These GWAS results suggested a possible protective effect of lower *GSTT1* copy number variants on the diol epoxide pathway, which was an unexpected result. Collectively, the results of this study provide further evidence that different patterns of cigarette smoking are responsible for the higher lung cancer risk of African Americans than of Whites and the lower lung cancer risk of Japanese Americans, while other factors appear to be involved in the differing risks of Native Hawaiians and Latinos.

## Introduction

Lung cancer is the leading cause of cancer death in both men and women in the U.S., with more than 158,000 deaths in 2015, approximately 90% of which are caused by cigarette smoking [1]. Worldwide in 2012 there were 1,589,800 deaths from lung cancer, about 3 per minute [2]. Cigarette smoking accounts for 80% of the worldwide lung cancer burden in males and at least 50% in females [3]. Diminishing this horrible death toll by science-based tobacco control policies and related approaches to prevention of lung cancer is a critical goal of cancer research. An understanding of the mechanisms by which smoking causes lung cancer is one approach to reach this goal.

Results from the Multiethnic Cohort (MEC) study provide a potentially important lead for understanding mechanisms of smoking-induced lung cancer. This study demonstrated that, for the same number of cigarettes smoked, and particularly at low levels of smoking, African-Americans and Native Hawaiians had a significantly higher risk for lung cancer than Whites while the risks of Latinos and Japanese-Americans were significantly lower than those of Whites [4]. Our hypothesis is that there are phenotypic and genotypic differences among these groups that could account for their differing risks for lung cancer. Thus, we have analyzed urine samples from subjects in each ethnic group for metabolites of carcinogens and toxicants which are found in tobacco smoke and have also carried out genome wide association studies (GWAS) in search of common genetic variants that might predict differences in levels of these metabolites [5–8]. The results to date demonstrate important differences in levels of metabolites of nicotine, the tobacco-specific lung carcinogen 4-(methylnitrosamino)-1-(3-pyridyl)-1-butanone (NNK), acrolein, crotonaldehyde, and benzene among the ethnic groups which may contribute to the observed differences in lung cancer susceptibility in the MEC [5–8]. We have also observed strong relationships between polymorphisms in certain genes–*CYP2A6*, *UGT2B10*, and *GSTT1* –and concentrations of specific metabolites [5–8].

**Fig 1:**
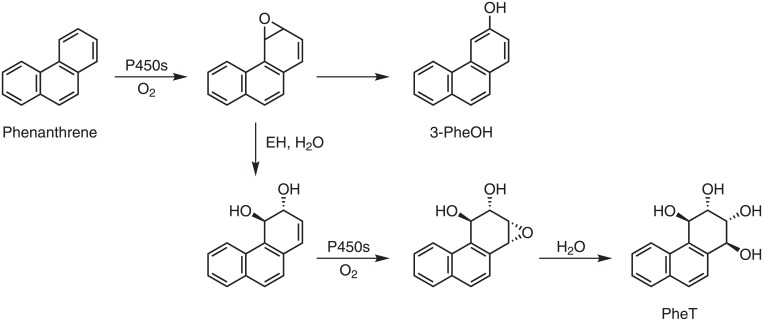
Metabolism of phenanthrene to 3-PheOH and PheT.

Polycyclic aromatic hydrocarbons (PAH) are a large group of structurally related compounds formed during the incomplete combustion and pyrolysis of organic matter. Common sources of exposure to PAH include polluted air, occupational exposures which occur in the aluminum and coke production industries and in roofing and paving with coal tar, consumption of broiled food, and cigarette smoking [9,10]. The planar PAH molecules, of which the most abundant have 3–5 aromatic rings, always occur together as mixtures. Many individual PAH demonstrate strong carcinogenic activity in the lung and other tissues of laboratory animals, and certain occupations associated with PAH exposure are considered carcinogenic to humans [9]. Benzo[*a*]pyrene, a widely studied prototypic and highly carcinogenic PAH, is also classified as carcinogenic to humans [9]. PAH are considered to be among the principal causes of lung cancer in cigarette smokers [11]. Phenanthrene, with 3 angular rings, is a typical member of the PAH class although it lacks significant carcinogenicity [9]. Phenanthrene metabolites have been widely used as dosimeters of PAH exposure because their levels vary with those of carcinogenic PAH metabolites and, due to the higher concentration of phenanthrene in PAH mixtures, phenanthrene metabolites are more readily quantified [12]. In the study reported here, we quantified 3-hydroxyphenanthrene (3-PheOH) and phenanthrene tetraol (PheT) (Fig 1) in the urine of smokers from the five different ethnic groups of the MEC, and carried out a GWAS to investigate genes associated with 3-PheOH and PheT levels in these smokers.

## Materials and Methods

### Subjects

Approval for this study, including the consent procedure, was obtained from the Institutional Review Boards of the University of Minnesota, the University of Hawaii, and the University of Southern California. Study participants provided written consent. IRB Code Number: 0912M75654. The subjects in this study are a subgroup of current smokers from the Multiethnic Cohort study (MEC). The MEC is a prospective cohort study investigating the association of genetic and lifestyle factors with chronic diseases in a population with diverse ethnic backgrounds [13]. The cohort consists of 215,251 men and women, ages 45 to 75 at baseline, belonging mainly to one of the following five ethnic/racial groups: African Americans, Native Hawaiians, Whites, Latinos, and Japanese Americans. Potential participants were identified and recruited between 1993 and 1996 in Hawaii and California (mainly Los Angeles County) through voter registration lists, drivers’ license files, and Health Care Financing Administration data. Each participant completed a mail-in self-administered questionnaire, which included questions regarding demographic, dietary, lifestyle, smoking, and other exposure factors.

Approximately 10 years after their entry into the MEC, 2,393 current smokers participated in the MEC bio-specimen sub-cohort where a blood sample and a first morning urine sample (subjects recruited in California) or overnight urine sample (subjects recruited in Hawaii) was collected. Participants also completed an epidemiologic questionnaire, smoking history questionnaire and medication record. The overnight urine sample was collected starting between 5 pm and 9 pm (depending on the subject) and includes all urine passed during the night as well as the first morning urine. All urine was kept on ice until processing. Aliquots were subsequently stored in a -80°C freezer until analysis. Approval for this study, including the consent procedure, was obtained from the Institutional Review Boards of the University of Minnesota, the University of Hawaii, and the University of Southern California.

Overnight or first morning urine was used to measure total nicotine equivalents (TNE), which includes the sum of nicotine and its metabolites nicotine glucuronide, cotinine, cotinine glucuronide, 3′-hydroxycotinine, 3′-hydroxycotinine glucuronide, and nicotine *N*-oxide [5], total 4-(methylnitrosamino)-1-(3-pyridyl)-1-butanol (NNAL)[7], *S*-phenylmercapturic acid (SPMA)[14], 3-hydroxypropylmercapturic acid (3-HPMA)[8], 3-hydroxy-1-methylpropylmercapturic acid (HMPMA)[8] as well as PheT and PheOH, for which the methods are described below. Creatinine was analyzed using a colorimetric microplate assay (CRE34-K01) purchased from Eagle Bioscience (http://stores.eaglebio.com/creatinine-microplate-assay-kit).

### Statistical methods

For this analysis, 2,310 subjects were retained. These subjects had TNE >1.27 nmol/ml (4-times the limit of quantitation) [5] and had either PheT or 3-PheOH measured. Of the 2,310 subjects, 14 subjects were missing measures of PheT and 56 subjects were missing measures of 3-PheOH.

Additionally, among the subjects retained for this analysis, participants who were missing measures of BMI or cigarettes per day (CPD) at the time of urine collection were imputed using the Markov Chain Monte Carlo method and PROC MI statement from the SAS v9.2 software (SAS institute, Cary, NC). Details regarding the imputation have been published [5,7].

To examine the correlation between PheT, 3-PheOH and measures of smoking [cigarettes per day (CPD) and TNE], Pearson’s partial correlation coefficients (r) were adjusted for age, sex and race/ethnicity and creatinine levels (natural log). The Wilcoxon Mann-Whitney test was used to compare the rank of PheT and 3-PheOH levels across race/ethnicity. Also, the covariate-adjusted geometric means for PheT and 3-PheOH were computed for each ethnic/racial group at the mean covariate vector. We used four multivariable linear models. The first adjusted for the following predictors: age at time of urine collection (continuous), sex (if applicable), race, BMI (natural log) and creatinine levels (natural log) and the second additionally adjusted for TNE. The remaining two models did not include creatinine levels. To better meet model assumptions, the measures of PheT and 3-PheOH were transformed by taking the natural log. For ease of interpretation, the values presented in the tables were back-transformed as geometric means to their natural scale.

### Analysis of 3-PheOH and PheT

These analyses were carried out essentially as described previously [15,16]. Certain modifications were made in the 3-PheOH assay to accommodate the large number of samples. The assay was carried out on 350 μL samples of urine, using 96-well plates. The first solid-phase extraction was performed on an Isolute (Biotage) 400 mg supported liquid-liquid extraction plate with elution by toluene. The samples were further purified on 96-well plates containing 30 mg per well Copper Phthalocyanine Rayon, then silylated and analyzed by GC-electron impact-MS as essentially previously described. Among blind duplicate samples, there was an inter-batch CV of 12.2% for PheT and 19.7% for 3-PheOH. The intra-batch CVs were 5.8% and 9.5% for the respective metabolites. Each batch contained approximately the same number of subjects from each sex and ethnic group.

### Genetic Methods

We genotyped a total of 2,418 current smokers using the Illumina Human1M-Duo BeadChip (1,199,187 SNPs), previously described elsewhere [6]. We also imputed variants in the 1000 Genomes Project (http://www.1000genomes.org/) using SHAPEIT [17] and IMPUTE2 [18] with a cosmopolitan reference panel with all groups included. Post imputation, SNPs were filtered with an IMPUTE2 info score ≥0.30 and minor allele frequency (MAF) >1% in any MEC ethnic group. For PheT and 3-PheOH, a total of 2,225 and 2,185, respectively, study participants with complete genotype and phenotype data, and 11,892,802 SNPs/indels (1,131,426 genotyped and 10,761,376 imputed) were included in the final analyses. The GWAS analyses were adjusted for age, sex, BMI, TNE, race and principal components, with a p-value cut-off of 5x10^-8^ for genome-wide significance. Among regions with multiple globally significant associations we used conditional models to determine the leading loci. We also performed ethnic-specific analyses to discover population specific loci of importance.

A *GSTT1* deletion polymorphism was successfully genotyped among 2,111 individuals via TaqMan, using a pre-designed TaqMan *GSTT1* copy number assay (Hs00010004), and run on the 7900HT Fast Real-Time System (Life Technologies, Foster City, CA). Copy number counts were calculated with the Life Technologies CopyCaller v2.0 software.

For the smoking variables, least-square means (or geometric means) were estimated and compared between populations, in this case, copies of *GSTT1*. R-squared values were used to assess percentage of variability of PheT and 3-PheOH accounted for by the variants examined.

## Results

Characteristics of the study subjects whose urine was analyzed for 3-PheOH or PheT, and information on their cigarette consumption and TNE are summarized in Table 1. There were 331–709 subjects per ethnic group, with mean ages ranging from 60–65 years, and BMI from 24.4–26.9 kg/m^2^. Creatinine ranged from a low of 54 mg/dL in Whites to a high of 89 mg/dL in African-Americans, and cigarettes per day from a low of 7.1 in Latinos to a high of 20 in Whites. TNE were lowest (27.2 nmol/mL) in Japanese Americans and highest (44.4 nmol/mL) in African Americans. All characteristics were significantly different among the groups (P<0.0001).

**Table 1:** Main characteristics of study participants^a^ stratified by race/ethnicity.

	African Americans		Native Hawaiians		Whites		Latinos		Japanese Americans		
	Median (Interquartile)										p-values
**All**		369		331		445		456		709	
**Age (years)**	64	(59–69)	60	(56–65)	62	(58–68)	65	(61–70)	62	(58–69)	<0.0001
**BMI (kg/m**^**2**^**)**	26.9	(23.4–30.6)	26.8	(24.1–30.8)	24.7	(22.0–28.0)	26.5	(24.1–29.9)	24.4	(21.9–27.0)	<0.0001
**Creatinine (mg/dL)**	89	(53–140)	60	(38–91)	54	(33–85)	77	(51–120)	55	(34–89)	<0.0001
**Cigarettes per day**	10	(5–15)	15	(8–20)	20	(10–20)	7.1	(4–12)	12	(10–20)	<0.0001
**TNE (nmol/mL)**	44.4	(26.8–73.8)	31.3	(20.0–48.5)	36.3	(21.9–61.5)	32.2	(20.9–53.6)	27.2	(15.8–43.5)	<0.0001
**Males**		112		120		193		239		404	
**Age (years)**	63	(58–66)	63	(58–68)	62	(59–67)	65	(62–71)	62	(58–68)	<0.0001
**BMI (kg/m**^**2**^**)**	26.3	(23.1–28.4)	26.8	(24.3–30.6)	25.8	(23.3–28.0)	25.8	(23.7–28.7)	24.8	(22.7–27.4)	<0.0001
**Creatinine (mg/dL)**	120	(81–170)	75	(51–120)	71	(48–110)	90	(58–140)	70	(44–110)	<0.0001
**Cigarettes per day**	10	(6.2–20)	19	(10–20)	20	(15–25)	10	(5–15)	15	(10–20)	<0.0001
**TNE (nmol/mL)**	54.0	(32.0–92.7)	33.4	(22.2–52.0)	42.4	(25.2–78.4)	34.6	(21.9–59.1)	30.0	(17.9–47.8)	<0.0001
**Females**		257		211		252		217		305	
**Age (years)**	64	(59–71)	59	(56–64)	62	(58–69)	64	(60–69)	62	(58–69)	<0.0001
**BMI (kg/m**^**2**^**)**	27.4	(23.5–31.5)	26.8	(23.9–31.1)	23.8	(21.0–27.8)	27.2	(24.2–30.7)	23.5	(20.7–26.5)	<0.0001
**Creatinine (mg/dL)**	79	(49–130)	54	(33–80)	47	(29–67)	65	(45–94)	43	(27–66)	<0.0001
**Cigarettes per day**	10	(5–15)	12	(8–20)	15	(7.5–20)	6	(4–10)	10	(7–15)	<0.0001
**TNE (nmol/mL)**	41.5	(25.9–66.1)	30.1	(19.1–46.0)	31.6	(20.3–50.7)	31.7	(18.3–50.1)	21.9	(13.6–35.3)	<0.0001

TNE, total nicotine equivalents

^a^ Includes all study participants with either PheT or 3-PheOH measures.

Males smoked significantly more cigarettes per day than females in all groups except African Americans (p = 0.08). TNE were significantly higher in males than in females among African Americans, Whites, and Japanese Americans (p’s < = 0.01).

Correlations among 3-PheOH, PheT, TNE, total NNAL, SPMA, 3-HPMA, and HMPMA are summarized in Table 2, for the entire group and separately for males and females. All correlations were statistically significant (most with P<0.0001).

**Table 2:** Pearson's r for metabolites of phenanthrene and other metabolites associated with cigarette smoking.

	n = 2028	All					n = 954	Males					n = 1074	Females				
	PheT	TNE	NNAL	SPMA	3-HPMA	HMPMA	PheT	TNE	NNAL	SPMA	3-HPMA	HMPMA	PheT	TNE	NNAL	SPMA	3-HPMA	HMPMA
PheT	ref	0.47	0.39	0.36	0.35	0.32	ref	0.47	0.39	0.37	0.3	0.31		0.46	0.39	0.34	0.38	0.33
p-value		<0.0001	<0.0001	<0.0001	<0.0001	<0.0001		<0.0001	<0.0001	<0.0001	<0.0001	<0.0001		<0.0001	<0.0001	<0.0001	<0.0001	<0.0001
3-PheOH	0.54	0.41	0.35	0.23	0.41	0.39	0.58	0.43	0.37	0.24	0.39	0.39	0.51	0.39	0.33	0.23	0.42	0.39
p-value	<0.0001	<0.0001	<0.0001	<0.0001	<0.0001	<0.0001	<0.0001	<0.0001	<0.0001	<0.0001	<0.0001	<0.0001	<0.0001	<0.0001	<0.0001	<0.0001	<0.0001	<0.0001
**African Americans**	n = 329						n = 104						n = 225					
PheT	ref	0.42	0.33	0.3	0.35	0.28		0.42	0.25	0.34	0.17	0.05	ref	0.42	0.37	0.29	0.43	0.39
p-value		<0.0001	<0.0001	<0.0001	<0.0001	<0.0001		<0.0001	<0.0001	<0.0001	0.02	0.05		<0.0001	<0.0001	<0.0001	<0.0001	<0.0001
3-PheOH	0.55	0.41	0.31	0.25	0.4	0.35	0.63	0.37	0.29	0.16	0.19	0.11	0.51	0.42	0.31	0.3	0.49	0.47
p-value		<0.0001	<0.0001	<0.0001	<0.0001	<0.0001	<0.0001	<0.0001	<0.0001	<0.0001	<0.0001	<0.0001	<0.0001	<0.0001	<0.0001	<0.0001	<0.0001	<0.0001
**Native Hawaiians**	n = 298						n = 111						n = 187					
PheT	ref	0.54	0.38	0.41	0.5	0.46		0.54	0.36	0.5	0.6	0.56	ref	0.55	0.4	0.36	0.45	0.41
p-value		<0.0001	<0.0001	<0.0001	<0.0001	<0.0001		<0.0001	<0.0001	<0.0001	<0.0001	<0.0001		<0.0001	<0.0001	<0.0001	<0.0001	<0.0001
3-PheOH	0.59	0.54	0.46	0.29	0.55	0.54	0.66	0.49	0.48	0.32	0.56	0.58	0.55	0.59	0.46	0.26	0.56	0.52
p-value	<0.0001	<0.0001	<0.0001	<0.0001	<0.0001	<0.0001	<0.0001	<0.0001	<0.0001	0.0006	<0.0001	<0.0001	<0.0001	<0.0001	<0.0001	<0.0001	<0.0001	<0.0001
**Whites**	n = 394						n = 174						n = 220					
PheT	ref	0.51	0.44	0.4	0.4	0.38		0.57	0.51	0.39	0.38	0.45	ref	0.48	0.4	0.4	0.41	0.34
p-value		<0.0001	<0.0001	<0.0001	<0.0001	<0.0001	<0.0001	<0.0001	<0.0001	<0.0001	<0.0001	<0.0001		<0.0001	<0.0001	<0.0001	<0.0001	<0.0001
3-PheOH	0.49	0.42	0.41	0.2	0.52	0.49	0.53	0.61	0.57	0.26	0.58	0.57	0.47	0.32	0.31	0.16	0.48	0.44
p-value	<0.0001	<0.0001	<0.0001	<0.0001	<0.0001	<0.0001	<0.0001	<0.0001	<0.0001	0.0006	<0.0001	<0.0001	<0.0001	<0.0001	<0.0001	<0.0001	<0.0001	<0.0001
**Latinos**	n = 402						n = 210						n = 192					
PheT	ref	0.4	0.36	0.45	0.23	0.21	ref	0.38	0.35	0.48	0.17	0.14		0.42	0.38	0.43	0.27	0.26
p-value		<0.0001	<0.0001	<0.0001	<0.0001	<0.0001		<0.0001	<0.0001	<0.0001	0.02	0.05		<0.0001	<0.0001	<0.0001	<0.0001	0.0003
3-PheOH	0.52	0.32	0.28	0.27	0.36	0.34	0.5	0.37	0.27	0.29	0.39	0.39	0.53	0.26	0.28	0.24	0.34	0.29
p-value	<0.0001	<0.0001	<0.0001	<0.0001	<0.0001	<0.0001	<0.0001	<0.0001	<0.0001	<0.0001	<0.0001	<0.0001	<0.0001	0.0002	<0.0001	<0.0001	<0.0001	<0.0001
**Japanese Americans**	n = 605						n = 355						n = 250					
PheT		0.51	0.44	0.27	0.4	0.39		0.5	0.44	0.27	0.39	0.41	ref	0.51	0.42	0.26	0.42	0.35
p-value		<0.0001	<0.0001	<0.0001	<0.0001	<0.0001		<0.0001	<0.0001	<0.0001	<0.0001	<0.0001		<0.0001	<0.0001	<0.0001	<0.0001	<0.0001
3-PheOH	0.56	0.41	0.32	0.16	0.46	0.42	0.6	0.39	0.31	0.14	0.48	0.43	0.5	0.43	0.33	0.2	0.45	0.41
p-value	<0.0001	<0.0001	<0.0001	<0.0001	<0.0001	<0.0001	<0.0001	<0.0001	<0.0001	<0.0001	<0.0001	<0.0001	<0.0001	<0.0001	<0.0001	<0.0001	0.002	<0.0001

PheT, phenanthrene tetraol; TNE, total nicotine equivalents; NNAL, 4-(methylnitrosamino)-1-(3-pyridyl)-1-butanol; SPMA, *S*-phenylmercapturic acid; 3-HPMA, 3-hydroxypropylmercapturic acid; HMPMA, 3-hydroxy-1-methylpropylmercapturic acid

The strongest correlations in the overall group were between 3-PheOH and PheT (Pearson’s r = 0.49–0.59).

Because of the large differences in creatinine levels among the groups, metabolite levels were expressed per mL urine. Medians and interquartile ranges for 3-PheOH and PheT are summarized in Table 3. The highest levels of 3-PheOH, 0.931 pmol/mL urine, were found in African Americans, and these were significantly higher (P<0.0001) than those in Whites, 0.697 pmol/mL urine. The lowest levels of 3-PheOH, 0.621 pmol/mL urine were found in Japanese Americans and these were significantly lower than in Whites (P = 4.50x10^-3^). High levels of PheT, 1.13 pmol/mL urine, were also found in African Americans, and were significantly higher than in Whites (0.853 pmol/mL urine, P = 0.0001). PheT levels in Japanese Americans were not significantly different from those in Whites. When dichotomized by sex, African Americans had higher levels of both 3-PheOH and PheT than Whites and Japanese Americans had lower levels than Whites, and most differences were significant.

**Table 3:** Median and interquartile range for measures of 3-PheOH and PheT.

	All				Males				Females			
	N	Median	(Interquartile range)	p-value when compared to Whites^a^	N	Median	(Interquartile range)	p-value when compared to Whites	N	Median	(Interquartile range)	p-value when compared to Whites
**3-PheOH (pmol/mL)**												
**African Americans**	358	0.931	(0.564–1.63)	<0.0001	110	1.18	(0.741–1.84)	4.00x10^-4^	248	0.814	(0.528–1.46)	<0.0001
**Native Hawaiians**	321	0.624	(0.426–0.888)	0.0107	117	0.680	(0.482–1.06)	0.0356	204	0.596	(0.385–0.831)	0.237
**Whites**	432	0.697	(0.437–1.08)		188	0.864	(0.488–1.38)		244	0.604	(0.402–0.916)	
**Latinos**	448	0.725	(0.463–1.15)	0.229	236	0.810	(0.536–1.42)	0.797	212	0.632	(0.400–0.989)	0.607
**Japanese Americans**	695	0.621	(0.414–0.922)	4.50x10^-3^	397	0.686	(0.468–1.01)	3.10x10^-3^	298	0.533	(0.350–0.803)	9.90x10^-3^
**p-value**			<0.0001				<0.0001				<0.0001	
**PheT (pmol/mL)**												
**African Americans**	367	1.13	(0.617–2.22)	1.00x10^-4^	112	1.40	(0.835–2.65)	0.0150	255	0.959	(0.565–2.05)	0.0001
**Native Hawaiians**	329	0.829	(0.484–1.35)	0.179	119	1.10	(0.707–1.64)	0.530	210	0.720	(0.420–1.19)	0.484
**Whites**	443	0.853	(0.506–1.64)		193	1.17	(0.639–2.31)		250	0.699	(0.446–1.40)	
**Latinos**	453	1.14	(0.694–1.86)	1.00x10^-4^	238	1.26	(0.788–2.07)	0.222	215	0.994	(0.579–1.64)	7.00x10^-4^
**Japanese Americans**	704	0.838	(0.503–1.39)	0.322	402	0.966	(0.605–1.67)	0.0477	302	0.672	(0.422–1.15)	0.356
**p-value**			<0.0001				<0.0001				<0.0001	

^a^ p-value using the Wilcoxon Mann-Whitney test.

Levels of 3-PheOH in the urine of Latinos were similar to those in Whites while levels in Native Hawaiians were significantly lower than in Whites (P = 0.0107). Levels of PheT in the urine of Latinos were significantly higher than in Whites (P<0.0001) while those of Native Hawaiians were similar to Whites. When dichotomized by sex, most of these comparisons were non-significant or borderline, except PheT in Latino females, which was significantly higher than in Whites (P = 7.00x10^-4^).

Geometric means of 3-PheOH and PheT, expressed per mL of urine, are presented in Table 4. In Model 1, adjusting for age, sex, and BMI, African Americans had the highest levels of both 3-PheOH and PheT and Japanese Americans the lowest; these levels were significantly different from those in Whites. In Model 2, additionally adjusting for TNE, similar results were obtained except that the Japanese Americans’ 3-PheOH levels were not significantly different from those of Whites, and their PheT levels were significantly higher than Whites. Latinos had significantly higher levels of 3-PheOH and PheT than Whites in both models while Native Hawaiians had lower levels than Whites, and these were significant for 3-PheOH in Model 1. Additional adjustment for creatinine in Model 1 abolished the significant difference between African Americans and Whites in levels of 3-PheOH and PheT (data not shown), due to the relatively high creatinine levels in African Americans (Table 1).

**Table 4:** Geometric means of 3-PheOH and PheT.

		Model 1^a^						Model 2^b^					
	N	Geometric means	(95% CI)					Geometric means	(95% CI)				
**3-PheOH (pmol/mL)**													
**African Americans**	358	1.06	(0.984–1.14)†			*	Ψ	0.848	(0.797–0.901)†		§	*	Ψ
**Native Hawaiians**	321	0.605	(0.559–0.654)†	‡		*		0.623	(0.585–0.664)	‡		*	Ψ
**Whites**	432	0.713	(0.667–0.762)	‡	§	*	Ψ	0.674	(0.639–0.712)	‡		*	
**Latinos**	448	0.784	(0.734–0.837)†	‡	§		Ψ	0.774	(0.734–0.816)†	‡	§		Ψ
**Japanese Americans**	695	0.593	(0.562–0.626)†	‡		*		0.675	(0.646–0.706)	‡	§	*	
**p-value**^c^			<0.0001						<0.0001				
**PheT (pmol/mL)**													
**African Americans**	367	1.30	(1.19–1.42)†			*	Ψ	0.992	(0.923–1.07)†		§	*	
**Native Hawaiians**	329	0.858	(0.782–0.941)	‡		*		0.888	(0.825–0.956)	‡		*	
**Whites**	443	0.927	(0.858–1.00)	‡		*	Ψ	0.870	(0.817–0.926)	‡		*	Ψ
**Latinos**	453	1.14	(1.05–1.23)†	‡	§		Ψ	1.12	(1.05–1.19)†	‡	§		Ψ
**Japanese Americans**	704	0.821	(0.771–0.875)†	‡		*		0.963	(0.916–1.01)†			*	
**p-value**^c^			<0.0001						<0.0001				

^a^ Model 1, adjusted for age, sex, and BMI

^b^ Model 2 additionally adjusted for TNE

^c^ Global p-value

^†^ Significant when compared to Whites

^‡^ Significant when compared to African Americans

^§^ Significant when compared to Native Hawaiians

* Significant when compared to Latinos

^Ψ^ Significant when compared to Japanese Americans

In the GWAS analyses of 3-PheOH and PheT, we observed little evidence of inflation in the test statistic in the overall multiethnic sample (λ = 1.0; S1 and S2 Figs) or in any single ethnic group (0.96 ≤ λ’s ≤ 1.0) for either phenotype. In the overall analysis for PheT, there were 408 globally significant variants located between 24.2–24.4 Mb near the region of *glutathione S-transferase T1* and *T2* (*GSTT1* and *GSTT2*) on chromosome 22q11 (S3 Fig, S1 Table). There were three other rare globally significant associations on chromosomes 1, 6 and 14. The chromosome 1 variant is located in the intronic region of an open reading frame of gene *C1orf159*, 109 Mb from the *GSTM1* gene. The chromosome 6 variant is located within gene *PPP1R14C*, a protein phosphatase gene involved in protein synthesis and metabolism, and the chromosome 14 variant is near gene *TTC6 / FOXA1*. As these three variants are rare, we focus our presentation on the globally significant associations on chromosome 22 near genes *GSTT1* and *GSTT2*.

When the 408 globally significant associations were conditioned on the *GSTT1* deletion polymorphism, there remained 262 globally significant variants; the *GSTT1* deletion does not fully explain the association with PheT (S2 Table). The *GSTT1* deletion explains only 2.2% of the variability in PheT, compared to the strongest association, rs5751777 (p = 1.8x10^-62^), which on its own explains 7.7% of the variability in PheT. The deletion allele, which is associated with significantly lower PheT levels in the overall group (p = 3.4 x 10^−16^), and in Native Hawaiians, Whites, and Latinos, varies in frequency across populations from 0.40 in Latinos to 0.66 in Japanese (Table 5). The highly significant associations observed at 22q11 are in part explained by the *GSTT1* deletion (n = 2,097; Table 5). Through forward selection regression analysis (at a threshold of 1x10^-3^) of the 262 variants that remained significantly associated with PheT after adjustment for the *GSTT1* deletion polymorphism, we identified 5 independent variants including our top association rs5751777 (Table 6). Together, these 5 variants explained 9.33% of the variability in PheT.

**Table 5:** Geometric means (95% CI) of PheT (pmol/mL) stratified by *GSTT1* copy number variants and race/ethnicity.

*GSTT1* copies	All			African Americans			Native Hawaiians			Whites			Latinos			Japanese Americans			
	N	Geometric means (95% CL)^a^		N	Geometric means (95% CL)^a^		N	Geometric means (95% CL)^a^		N	Geometric means (95% CL)^a^		N	Geometric means (95% CL)^a^		N	Geometric means (95% CL)^a^		p-value
**0**	614	0.83	(0.79–0.88)	75	1.18	(0.98–1.41)^b^	75	0.71	(0.62–0.80)	77	0.72	(0.63–0.83)	87	0.76	(0.66–0.89)	300	0.79	(0.74–0.85)^b^	9.6 x 10^−4^
**1**	963	0.95	(0.91–0.99)	174	1.25	(1.11–1.40)^b^	138	0.86	(0.79–0.95)	202	0.86	(0.79–0.94)	185	1.07	(0.97–1.2)^b^	265	0.84	(0.79–0.91)^b^	7.5 x 10^−6^
**2**	520	1.16	(1.09–1.22)	87	1.28	(1.08–1.51)	65	1.03	(0.90–1.17)	142	1.17	(1.06–1.30)	162	1.37	(1.2–1.52)^b^	64	0.93	(0.81–1.07)	4.6 x 10^−4^
**p-value**	3.4x10^-16^			0.784			3.6x10^-4^			2.0x10^-7^			5.1x10^-9^			0.089			
**Null frequency**				0.47			0.51			0.41			0.4			0.66			
**Variation explained**	2.20%			0.10%			2.87%			4.20%			5.70%			0.38%			

^a^ Adjusted for age, sex, BMI, and TNE

^b^ P-value <0.05 when compared to Whites

**Table 6:** Five significant variants remaining from stepwise regression analysis.

					Alternate allele frequency in 1000 genomes							
Chr^a^	SNP^b^	BP	Reference allele	Alternate allele	African samples	American samples	Asian samples	European samples	Beta^c^	P^d^	Type^e^	Gene^f^
22	rs5751777	24267047	C	T	0.4533	0.5166	0.5087	0.6174	0.3117	1.31E-45	intergenic	*GSTT2*
22	rs1006771	24314006	G	T	0.6565	0.6326	0.5245	0.6649	0.2116	1.41E-23	UTR3	*DDTL*
22	rs80020284	24312589	G	A	0.2642	0.1188	0.0122	0.0488	-0.3459	6.61E-17	intronic	*DDTL*
22	rs2154595	24268647	C	T	0.2297	0.0608	0	0.0699	-0.3179	2.21E-12	intergenic	*GSTT2*
22	rs35921388	24401503	C	T	0.0996	0.2182	0.3462	0.223	-0.1912	2.85E-11	ncRNA_exonic	*GSTTP2*

^a^ Chromosome

^b^ Base pair, chromosomal position

^c^ Effect estimate per alternate allele carried

^d^ p-value from test of association

^e^ SNP variant/classification

^f^ Nearest gene designation

In ethnic specific GWAS analyses for African Americans there were 16 globally significant variants on chromosome 22 near the *GSTT2* gene, for Latinos there were 141 globally significant variants, for Japanese Americans there were 206 globally significant variants, for both the Native Hawaiians and Whites there were 59 globally significant variants near the *GSTT2* and *GSTTP1* genes (S4–S8 Figs). The *GSTT1* deletion polymorphism explained from 0.1% of variability in PheT among African Americans to 5.7% of the variability in PheT among Latinos (Table 5). Increasing *GSTT1* copy numbers were significantly associated with higher PheT levels (p = 3.4 x 10^−16^) in the overall group, and in Native Hawaiians, Whites, and Latinos (Table 5).

There were no globally significant variants for either the overall or the ethnic specific analyses for 3-PheOH using our genomic threshold of p <5x10^-8^ (S2 Fig).

## Discussion

In previous analyses of tobacco smoke carcinogen and toxicant metabolites in urine samples from the MEC, we have observed that African Americans had the highest TNE levels and Japanese Americans the lowest, both being significantly different from the intermediate levels in Whites [5]. Consistent with these observations, African Americans also had the highest levels of NNAL and its glucuronides, metabolites of the tobacco-specific lung carcinogen NNK, while Japanese Americans had the lowest, and these were significantly different from those in Whites [7]. Further studies demonstrated completely analogous results for *S*-phenylmercapturic acid, a metabolite of the volatile toxicant and carcinogen benzene [14]. In the study reported here, we have extended these analyses to 3-PheOH and PheT, metabolites of phenanthrene, a representative PAH. Consistent with the previous studies, we find high levels of 3-PheOH and PheT in African Americans, and the lowest level of 3-PheOH in Japanese Americans, and these were significantly different from those in Whites. Overall, these results provide compelling evidence that exposure to representative tobacco smoke carcinogens—NNK, benzene, and PAH—is highest in African Americans, who are at highest risk for lung cancer in the MEC, and lowest in Japanese Americans, at lowest risk for lung cancer. Thus, differences in carcinogen exposure from cigarette smoke among these three groups can at least partially explain their differing risks for lung cancer. African Americans smoke cigarettes differently from Whites, extracting more nicotine and more carcinogens per cigarette (Table 1 and [5,7]). This is also consistent with the observation that, among African Americans, the time to first cigarette upon waking in the morning is significantly shorter than in Whites [19]. Japanese Americans have a higher number of *CYP2A6* polymorphisms associated with lower nicotine metabolism than Whites, and consequently need to obtain less nicotine per cigarette and are therefore exposed to lower amounts of other toxicants and carcinogens than Whites [20], although that effect was not so strong in this study, perhaps due to other PAH exposure routes in Japanese Americans.

Hydroxylated PAH such as urinary 3-PheOH and the closely related compound 1-hydroxypyrene are accepted biomarkers of PAH exposure [21]. These simple hydroxylated PAH are metabolically formed by a cytochrome P450-catalyzed epoxidation of the PAH ring followed by rearrangement of the epoxide (“the NIH shift”) or by direct oxidation of the PAH ring [9,10]. These simple metabolic processes favor a direct relationship between PAH exposure and level of the hydroxylated metabolite in urine. Furthermore, in the case of phenanthrene, approximately 90% of its excreted metabolites are found in urine, based on studies in rats [22]. Thus urinary 3-PheOH is an excellent biomarker of exposure to phenanthrene, a representative PAH closely related to carcinogenic PAH such as benz[*a*]anthracene, chrysene, benzofluoranthenes, and benzo[*a*]pyrene which are simultaneously formed during cigarette smoking by combustion of tobacco. The NHANES study of 3-PheOH reported levels of this urinary metabolite in smokers that are similar to those in our study [12]. The metabolism pathway leading to PheT is more complex, and interpretation of the resulting data might not be as straightforward [23]. The initially formed epoxide is hydrated to a dihydrodiol, catalyzed by epoxide hydrolase. This dihydrodiol is then further oxidized to a dihydrodiol epoxide, which then undergoes hydrolysis to form PheT. As discussed below, the epoxides potentially can be detoxified by glutathione-*S*-transferases, which could influence PheT levels. For higher PAH such as benzo[*a*]pyrene, the same sequence of reactions resulting in PheT leads to a major ultimate carcinogen, benzo[*a*]pyrene-7,8-dihydrodiol-9,10-epoxide (BPDE) [10,24,25]. Thus, PheT can be considered a surrogate for PAH exposure *plus* metabolic activation, and levels of PheT in human urine correlate with those of the tetraol derived from BPDE [26]. In spite of these complexities, exposure to phenanthrene is a major driving force for PheT formation, as indicated by the correlation between 3-PheOH and PheT (Pearson’s r = 0.49–0.59).

Considerable evidence favors a significant role for PAH as causes of lung cancer in cigarette smokers [27]. Multiple PAH have been identified in cigarette smoke and some of these are powerful carcinogens, readily inducing tumors at the site of application, including the lung, as well as at distant sites [11,28,29]. Subfractions of cigarette smoke condensate enriched in PAH are tumor initiators on mouse skin, and the combination of cigarette smoke PAH and tumor promoters or co-carcinogens partially recapitulates the carcinogenic activity of cigarette smoke condensate in mouse skin experiments [28]. Total levels of established lung carcinogenic PAH in cigarette smoke are about 50–60 ng/cigarette, similar to levels of the lung carcinogen NNK [27]. DNA adducts of BPDE have been identified in lung tissue from some smokers, and reactions of BPDE and other PAH diol epoxide metabolites with the *p53* tumor suppressor gene produce adducts at the same sites known to be mutational hot spots in human lung cancer (although the same pattern is also formed by acrolein) [30–33].

Levels of 3-PheOH were significantly lower in Native Hawaiians than in Whites, which is consistent with previous observations for TNE and total NNAL. These results suggest that intake of smoke constituents in Native Hawaiians is less than in Whites, in spite of the fact that they are at higher risk for lung cancer. We have reported however that urinary levels of the acrolein biomarker 3-HPMA were highest in Native Hawaiians compared to the other MEC groups, suggesting that endogenous processes related to lipid peroxidation perhaps play a role in the relatively high risk of Native Hawaiians for lung cancer. Levels of PheT were significantly higher in Latinos than in Whites, and this effect was confined to females. This is different from our observations with TNE and total NNAL, and requires further study. Collectively, these observations suggest that there are factors influencing lung cancer susceptibility in Native Hawaiians and Latinos that have not been recognized in our metabolic and genetic studies to date.

The GWAS demonstrated a signal associated with PheT on chromosome 22 near *GSTT1* and *GSTT2*. While glutathione S-transferases M1 (GSTM1), GSTP1, and GSTA1 are known to catalyze the detoxification of diol epoxides formed from various PAH, there is scant evidence that GSTT1 or GSTT2 have activity in the detoxification of PAH epoxides or diol epoxides, including those formed from phenanthrene [34–39]. To the extent that such activity exists, we would have expected *lower* levels of PheT in individuals with both copies of the *GSTT1* gene because the diol epoxide precursor to PheT would be intercepted (Fig 1), but instead we observed significantly *higher* levels of PheT in individuals with both copies of the gene (Table 5). There is evidence from some previous studies that the *GSTT1* deletion is protective, which would be consistent with our surprising observation of lower PheT levels in the null individuals, but this requires further study [40]. Apparently, the relationship of GSTT1 and GSTT2 activity to PheT levels may be more complex than previously thought.

In summary, the results of this study demonstrate higher uptake of carcinogenic PAH, as demonstrated by urinary 3-PheOH and PheT levels, in African American smokers than in Whites and lower levels in Japanese American smokers. These observations are consistent with our previous studies demonstrating patterns of nicotine, tobacco-specific nitrosamine, and benzene uptake in these groups, consistent with their differing risks for lung cancer. The relationships of PAH uptake to lung cancer risk in Native Hawaiians and Latinos appear to be more complex. We also observed strong effects of *GSTT1* deletion and a *GSTT2* polymorphism on PheT levels, suggesting previously unknown factors influencing the PAH diol epoxide metabolic activation pathway.

## Supporting Information

S1 Fig Quantile-Quantile plot of observed and expected –log_10_ transformed p-values from association between PheT levels and genotyped or imputed alleles from the multiethnic GWAS analysis. (JPG)

S2 Fig Quantile-Quantile plot of observed and expected –log_10_ transformed p-values from association between 3-PheOH levels and genotyped or imputed alleles from the multiethnic GWAS analysis. (JPG)

S3 Fig Locus Zoom plot of *GSTT2/GSTT1* in our multi-ethnic sample with European LD values. (JPG)

S4 Fig Locus Zoom plot of *GSTT2/GSTT1* in our African American sample with European LD values. (JPG)

S5 Fig Locus Zoom plot of *GSTT2/GSTT1* in our Latino sample with European LD values. (JPG)

S6 Fig Locus Zoom plot of *GSTT2/GSTT1* in our Japanese American sample with European LD values. (JPG)

S7 Fig Locus Zoom plot of *GSTT2/GSTT1* in our Native Hawaiian sample with European LD values. (JPG)

S8 Fig Locus Zoom plot of *GSTT2/GSTT1* in our White sample with European LD values. (JPG)

S1 Table List of 412 globally significant associations (p < 5E-8) for PheT in the MEC Cohort. (CSV)

S2 Table List of 262 globally significant associations (p < 5E-8) after conditioning on the GSTT1 deletion polymorphism. (CSV)

## References

[1] Siegel RL, Miller KD, Jemal A. Cancer statistics, 2015. CA Cancer J Clin. 2015;65: 5–29. 10.3322/caac.21254 25559415

[2] International Agency for Research on Cancer.The Global and Regional Burden of Cancer In: Stewart BW, Wild CP, editors. World Cancer Report 2014. Lyon, FR: International Agency for Research on Cancer; 2014 pp. 16–53.

[3] Jemal A, Bray F, Center MM, Ferlay J, Ward E, Forman D. Global cancer statistics. CA Cancer J Clin. 2011;61: 69–90. 10.3322/caac.20107 21296855

[4] Haiman CA, Stram DO, Wilkens LR, Pike MC, Kolonel LN, Henderson BE, et al Ethnic and racial differences in the smoking-related risk of lung cancer. N Engl J Med. 2006;354: 333–342. 16436765 10.1056/NEJMoa033250

[5] Murphy SE, Park S-SL, Thompson EF, Wilkens LR, Patel Y, Stram DO, et al Nicotine *N*-glucurionidation relative to *N*-oxidation and *C*-oxidation and UGT2B10 genotype in five ethnic/racial groups. Carcinogenesis. 2014;35: 2526–2533. 10.1093/carcin/bgu191 25233931 PMC4216060

[6] Patel Y, Stram DO, Wilkens LR, Park SL, Henderson BE, Le Marchand L, et al The contribution of common genetic variation to nicotine and cotinine glucuronidation in multiple ethnic/racial populations. Cancer Epidemiol Biomarkers Prev. 2015;24: 119–127. 10.1158/1055-9965.EPI-14-0815 25293881 PMC4294952

[7] Park SL, Carmella SG, Ming X, Stram DO, Le Marchand L, Hecht SS. Variation in levels of the lung carcinogen NNAL and its glucuronides in the urine of cigarette smokers from five ethnic groups with differing risks for lung cancer. Cancer Epidemiol Biomarkers Prev. 2015;24: 561–569. 10.1158/1055-9965.EPI-14-1054 25542827 PMC4355389

[8] Park SL, Carmella SG, Chen M, Patel Y, Stram DO, Haiman CA, et al Mercpaturic acids derived from the toxicants acrolein and crotonaldehyde in the urine of cigarettes smokers from five ethnic groups with differing risks for lung cancer. PLoS One. 2015;10: e0124841 10.1371/journal.pone.0124841 26053186 PMC4460074

[9] International Agency for Research on Cancer. Some Non-Heterocyclic Polycyclic Aromatic Hydrocarbons and Some Related Exposures IARC Monographs on the Evaluation of Carcinogenic Risks to Humans, v. 92 Lyon, FR: IARC; 2010 pp. 35–818. PMC4781319 21141735

[10] Dipple A, Moschel RC, Bigger CAH. Polynuclear aromatic hydrocarbons In: Searle CE, editor. Chemical Carcinogens, Second Edition, ACS Monograph 182 vol. 1 Washington, D.C.: American Chemical Society; 1984 pp. 41–163.

[11] Hecht SS. Tobacco smoke carcinogens and lung cancer. J Natl Cancer Inst. 1999;91: 1194–1210. 10413421 10.1093/jnci/91.14.1194

[12] Suwan-ampai P, Navas-Acien A, Strickland PT, Agnew J. Involuntary tobacco smoke exposure and urinary levels of polycyclic aromatic hydrocarbons in the United States, 1999 to 2002. Cancer Epidemiol Biomarkers Prev. 2009;18: 884–893. 10.1158/1055-9965.EPI-08-0939 19258471

[13] Kolonel LN, Henderson BE, Hankin JH, Nomura AM, Wilkens LR, Pike MC, et al A multiethnic cohort in Hawaii and Los Angeles: baseline characteristics. Am J Epidemiol. 2000;151: 346–357. 10695593 10.1093/oxfordjournals.aje.a010213 PMC4482109

[14] Haiman CA, Patel YM, Stram DO, Carmella SG, Chen M, Wilkens L, et al Benzene uptake and glutathione S-transferase T1 status as determinants of S-phenylmercapturic acid in cigarette smokers in the Multiethnic Cohort. PLoS One. 2015. 10.1371/journal.pone.0150641 PMC4784986 26959369

[15] Carmella SG, Ming X, Olvera N, Brookmeyer C, Yoder A, Hecht SS. High throughput liquid and gas chromatography-tandem mass spectrometry assays for tobacco-specific nitrosamine and polycyclic aromatic hydrocarbon metabolites associated with lung cancer in smokers. Chem Res Toxicol. 2013;26: 1209–1217. 10.1021/tx400121n 23837805 PMC3803150

[16] Carmella SG, Chen M, Yagi H, Jerina DM, Hecht SS. Analysis of phenanthrols in human urine by gas chromatography-mass spectrometry: potential use in carcinogen metabolite phenotyping. Cancer Epidemiol Biomarkers Prev. 2004;13: 2167–2174. 15598776

[17] Delaneau O, Marchini J, Zagury JF. A linear complexity phasing method for thousands of genomes. Nat Methods. 2012;9: 179–181. 10.1038/nmeth.1785 22138821

[18] Howie BN, Donnelly P, Marchini J. A flexible and accurate genotype imputation method for the next generation of genome-wide association studies. PLoS Genet. 2009;5: e1000529 10.1371/journal.pgen.1000529 19543373 PMC2689936

[19] Branstetter SA, Mercincavage M, Muscat JE. Predictors of the nicotine dependence behavior time to the first cigarette in a multiracial cohort. Nicotine Tob Res. 2015;17: 819–824. 10.1093/ntr/ntu236 25431372 PMC4481692

[20] Park S-L, Tiirikainen M, Patel Y, Wilkens LR, Stram DO, Le Marchand L, et al Genetic determinants of CYP2A6 activity across racial/ethnic groups with different risk of lung cancer and effect on their smoking behavior. Carcinogenesis. 2016;in press. 10.1093/carcin/bgw012 PMC5014092 26818358

[21] Hecht SS. Human urinary carcinogen metabolites: biomarkers for investigating tobacco and cancer. Carcinogenesis. 2002;23: 907–922. 12082012 10.1093/carcin/23.6.907

[22] Chu I, Ng KM, Benoit FM, Moir D. Comparative metabolism of phenanthrene in the rat and guinea pig. J Environ Sci Health, Part B. 1992;27: 729–749. 10.1080/03601239209372809 1460244

[23] Hecht SS, Chen M, Yagi H, Jerina DM, Carmella SG. *r*-1,*t*-2,3,*c*-4-Tetrahydroxy-1,2,3,4-tetrahydrophenanthrene in human urine: a potential biomarker for assessing polycyclic aromatic hydrocarbon metabolic activation. Cancer Epidemiol Biomarkers Prev. 2003;12: 1501–1508. 14693744

[24] Cooper CS, Grover PL, Sims P. The metabolism and activation of benzo[a]pyrene. Prog Drug Metab. 1983;7: 295–396.

[25] Conney AH. Induction of microsomal enzymes by foreign chemicals and carcinogenesis by polycyclic aromatic hydrocarbons: G.H.A. Clowes Memorial Lecture. Cancer Res. 1982;42: 4875–4917. 6814745

[26] Hochalter JB, Zhong Y, Han S, Carmella SG, Hecht SS. Quantitation of a minor enantiomer of phenanthrene tetraol in human urine: correlations with levels of overall phenanthrene tetraol, benzo[*a*]pyrene tetraol, and 1-hydroxypyrene. Chem Res Toxicol. 2011;24: 262–268. 10.1021/tx100391z 21229973 PMC3076645

[27] Hecht SS. Tobacco smoke carcinogens and lung cancer In: Penning TM, editor. Chemical Carcinogenesis. Springer; 2011 pp. 53–74.

[28] Hoffmann D, Schmeltz I, Hecht SS, Wynder EL. Tobacco carcinogenesis In: Gelboin H, Ts'o POP, editors. Polycyclic Hydrocarbons and Cancer. New York: Academic Press; 1978 pp. 85–117.

[29] Rodgman A, Perfetti T. The Chemical Components of Tobacco and Tobacco Smoke. Boca Raton, FL: CRC Press; 2009.

[30] Denissenko MF, Pao A, Tang M, Pfeifer GP. Preferential formation of benzo[*a*]pyrene adducts at lung cancer mutational hot spots in P53. Science. 1996;274: 430–432. 8832894 10.1126/science.274.5286.430

[31] Smith LE, Denissenko MF, Bennett WP, Li H, Amin S, Tang M, et al Targeting of lung cancer mutational hotspots by polycyclic aromatic hydrocarbons. J Natl Cancer Inst. 2000;92: 803–811. 10814675 10.1093/jnci/92.10.803

[32] Tretyakova N, Matter B, Jones R, Shallop A. Formation of benzo[*a*]pyrene diol epoxide-DNA adducts at specific guanines within *K-ras* and *p53* gene sequences: stable isotope-labeling mass spectrometry approach. Biochemistry. 2002;41: 9535–9544. 12135376 10.1021/bi025540i

[33] Feng Z, Hu W, Hu Y, Tang M-S. Acrolein is a major cigarette-related lung cancer agent. Preferential binding at *p53* mutational hotspots and inhibition of DNA repair. Proc Natl Acad Sci USA. 2006;103: 15404–15409. 17030796 10.1073/pnas.0607031103 PMC1592536

[34] Upadhyaya P, Hochalter JB, Balbo S, McIntee EJ, Hecht SS. Preferential glutathione conjugation of a reverse diol epoxide compared with a bay region diol epoxide of benzo[a]pyrene in human hepatocytes. Drug Metab Dispos. 2010;38: 1397–1402. 10.1124/dmd.110.034181 20547966 PMC2939474

[35] Hecht SS, Berg JZ, Hochalter JB. Preferential glutathione conjugation of a reverse diol epoxide compared to a bay region diol epoxide of phenanthrene in human hepatocytes: Relevance to molecular epidemiology studies of glutathione-*S*-transferase polymorphisms and cancer. Chem Res Toxicol. 2009;22: 426–432. 10.1021/tx800315m 19187038 PMC2765539

[36] Rojas M, Cascorbi I, Alexandrov K, Kriek E, Auburtin G, Mayer L, et al Modulation of benzo[*a*]pyrene diolepoxide-DNA adduct levels in human white blood cells by CYP1A1, GSTM1, and GSTT1 polymorphims. Carcinogenesis. 2000;21: 35–41. 10607731 10.1093/carcin/21.1.35

[37] Sundberg K, Dreij K, Seidel A, Jernström B. Glutathione conjugation and DNA adduct formation of dibenzo[*a*,*l*]pyrene and benzo[a]pyrene diol epoxides in V79 cells stably expressing different human glutathione transferases. Chem Res Toxicol. 2002;15: 170–179. 11849043 10.1021/tx015546t

[38] Fields WR, Morrow CS, Doss AJ, Sundberg K, Jernstrom B, Townsend AJ. Overexpression of stably transfected human glutathione S-transferase P1-1 protects against DNA damage by benzo[a]pyrene diol-epoxide in human T47D cells. Mol Pharmacol. 1998;54: 298–304. 9687571 10.1124/mol.54.2.298

[39] Landi S. Mammalian class theta GST and differential susceptibility to carcinogens: a review. Mutat Res. 2000;463: 247–283. 11018744 10.1016/s1383-5742(00)00050-8

[40] Garte S, Taioli E, Popov T, Kalina I, Sram R, Farmer P. Role of GSTT1 deletion in DNA oxidative damage by exposure to polycyclic aromatic hydrocarbons in humans. Int J Cancer. 2007;120: 2499–2503. 17330842 10.1002/ijc.22477

