# Supplementary figures and images for: Metabolites of the Polycyclic Aromatic Hydrocarbon Phenanthrene in the Urine of Cigarette Smokers from Five Ethnic Groups with Differing Risks for Lung Cancer
Source: PLoS One. 2016 Jun 8;11(6):e0156203. doi: 10.1371/journal.pone.0156203 (PMC4898721; doi:10.1371/journal.pone.0156203)

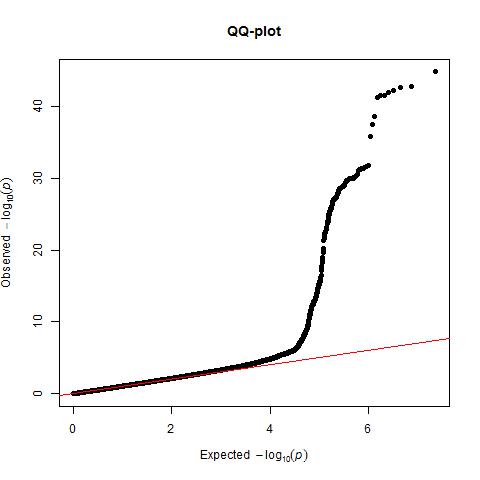

Supplement: S1 Fig — (JPG) [file pone.0156203.s001.jpg]

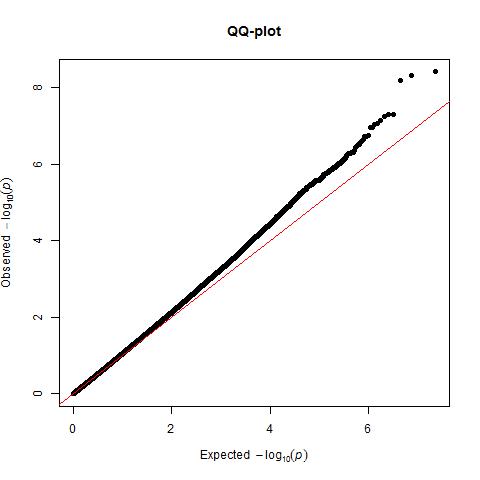

Supplement: S2 Fig — (JPG) [file pone.0156203.s002.jpg]

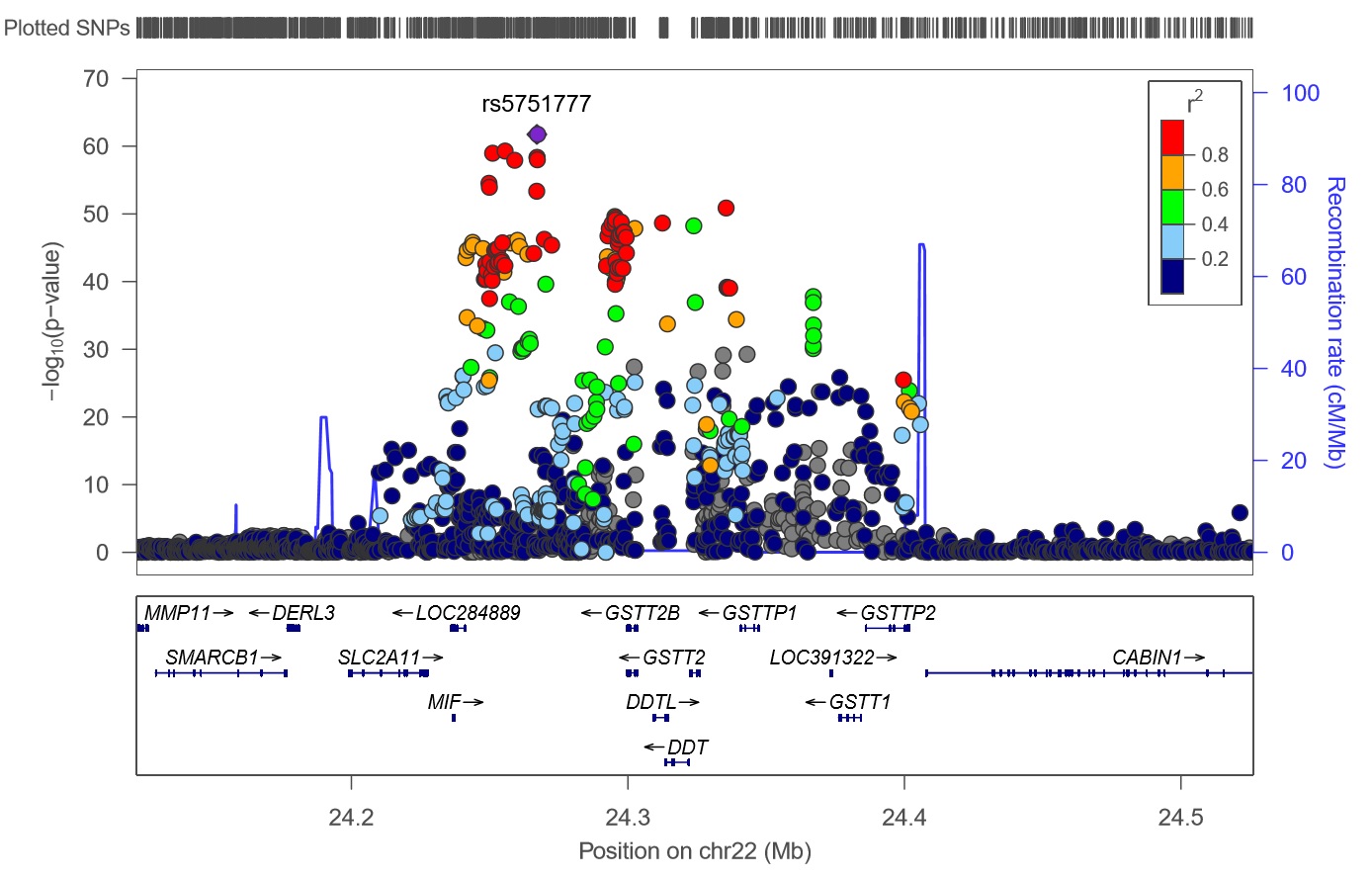

Supplement: S3 Fig — (JPG) [file pone.0156203.s003.jpg]

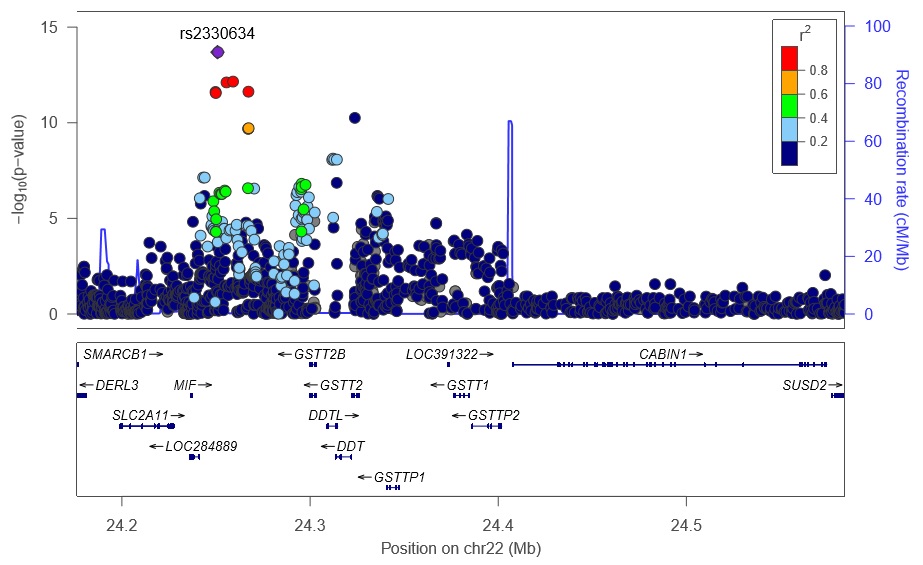

Supplement: S4 Fig — (JPG) [file pone.0156203.s004.jpg]

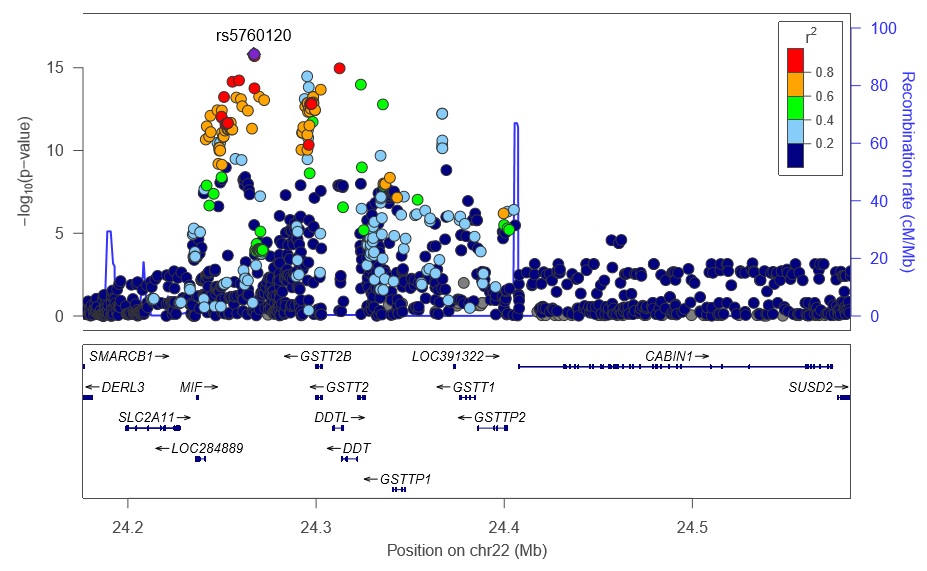

Supplement: S5 Fig — (JPG) [file pone.0156203.s005.jpg]

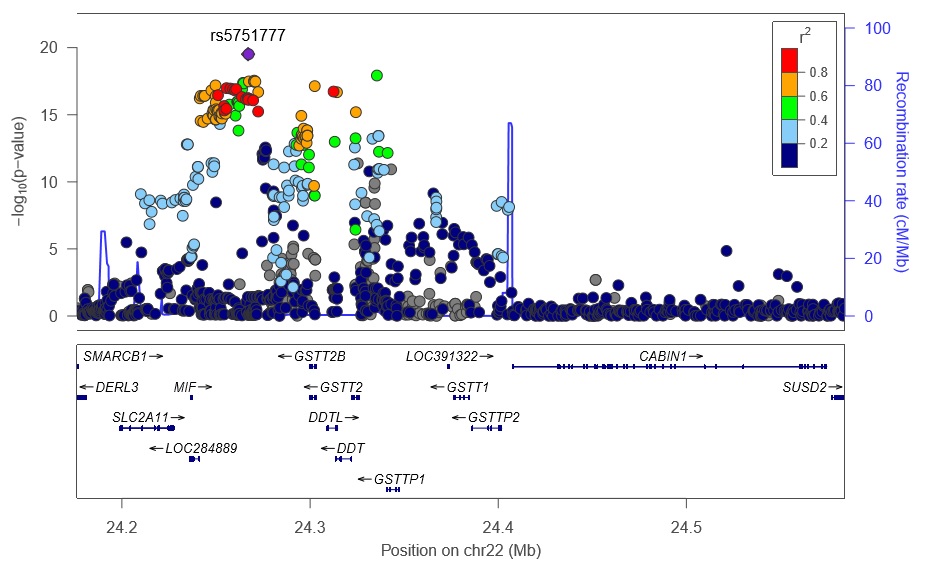

Supplement: S6 Fig — (JPG) [file pone.0156203.s006.jpg]

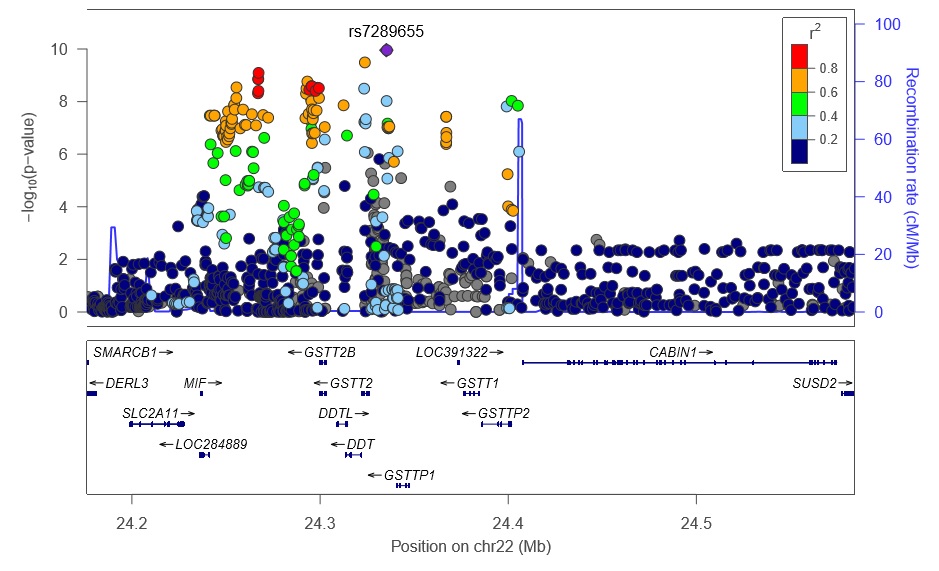

Supplement: S7 Fig — (JPG) [file pone.0156203.s007.jpg]

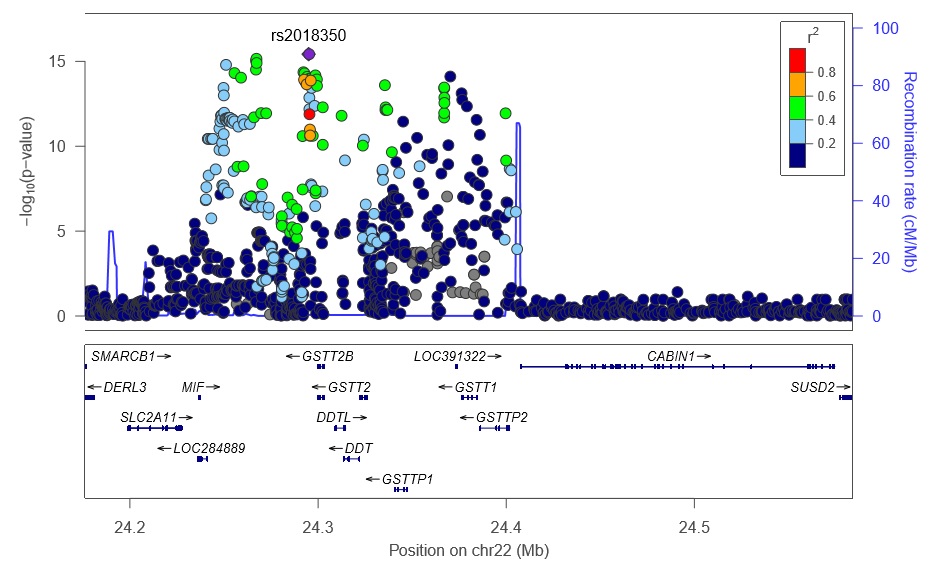

Supplement: S8 Fig — (JPG) [file pone.0156203.s008.jpg]
